# Effects of Bisphenol A Exposure during Pregnancy and lactation on Hippocampal Function in Newborn Rats

**DOI:** 10.7150/ijms.47300

**Published:** 2020-07-06

**Authors:** Ying Wang, Xiaomin Du, Dan Wang, Jun Wang, Juan Du

**Affiliations:** Department of Obstetrics and Gynecology, Shengjing Hospital of China Medical University, Shenyang, Liaoning, P.R. China.

**Keywords:** Bisphenol A, hippocampal function, Rho A, Rac1, apoptosis

## Abstract

Bisphenol A (BPA) is widely used in industrial production. It is closely related to the growth and development of the nervous system, and can enter the fetal circulation through the placental barrier, and can be secreted through breast milk. The development of nervous system is very important in fetus and neonatal period. The purpose of this study is to investigate the effects of different doses of BPA on learning and memory function of nervous system in rats. Pregnant rats were randomly divided into three treatment groups (control group, 5 mg/kg/d, 50 mg/kg/d). All animals received BPA from the discovery of pregnancy to 21 days after birth. Results had shown that after high concentration BPA exposure, the increase of PS amplitude and f-EPSP slope in hippocampal CA1 area of male offspring was lower than that of control group. High concentration of BPA could inhibit Nestin, Cyclin D1, bcl-2 and Rac1 in male offspring rats and the expression of bax and RhoA was promoted by BPA. In summary, our study indicated that BPA exposure during pregnancy and lactation could impair the hippocampal function of male offspring by affecting the growth and apoptosis of hippocampal neurons, which might be due to the abnormal regulation of RhoA and Rac1.

## Introduction

Bisphenol A (BPA) exerts estrogen-like activity by interacting with the classical estrogen receptors (ERα and ERβ) or the G protein-coupled receptor (GPR30) 1. Recent studies have shown that prenatal and neonatal exposure to BPA in low dose is linked to a wide variety of negative effects, including defects in male and female reproductive tracts, meiotic abnormalities in fetal oocytes, and complications during pregnancy 2. However, the mechanism is still unclear.

BPA is synthesized by condensation of two molecules of phenol and one molecule of acetone. Its chemical structure is similar to that of estradiol (E2), which has estrogenic effect and can bind and activate estrogen receptors (ERs) 3. Although the binding capacity of BPA to estrogen receptor is only 1/10 000 of that of E2, it can still cause early puberty, ovarian cycle disorder and abnormal embryonic development in female mammals 4. Epidemiological investigations and related studies indicate that BPA levels in women's blood are closely related to obesity, polycystic ovarian syndrome, recurrent abortion and endometrial hyperplasia 5. These evidences suggest that BPA, as an environmental endocrine disruptor, can affect the developmental function of pregnant and even offspring. Many animal behavioral tests showed that perinatal exposure to bisphenol A could affect the growth and development of offspring 6, and the mechanism of these behavioral changes is still unclear. We intend to study the specific mechanism of perinatal exposure to bisphenol A affecting the growth and development of offspring.

Synaptic plasticity mainly includes short-term synaptic plasticity and long-term synaptic plasticity, which have been recognized as the biological basis of learning and memory activities at the cellular level. Detection of synaptic plasticity in hippocampus by electrophysiological experiments is considered to be an important index for observing the changes of hippocampal function 7. Long-term exposure to toxicants can damage nerve morphology, inhibit the proliferation of neurons and induce neuronal apoptosis 8. Decreased proliferation and excessive apoptosis of these normal neurons may contribute to the impairment of hippocampal function 7. In addition, the formation of normal hippocampal function depends on the normal development of dendrites 9. When exploring the role of BPA, the researchers regard hippocampus as an important detection direction.

The Rho subfamily of small G proteins, including RhoA and Rac1, are important cytoskeleton regulators of actin and play an important role in tree burst development. Studies have shown that RhoA and Rac1 play an important regulatory role in neuronal dendritic development 10, 11. Rac1 positively regulates the growth and branching of dendrites, while RhoA inhibits the growth and branching of dendrites 12, 13. In the process of dendrite formation and elongation, Rac1 activity is higher while RhoA activity is lower 14. However, there are few studies on the regulatory effects of BPA on RhoA and Rac1.

In this study, the effect of BPA on the hippocampal function of offspring rats was observed by applying BPA to pregnant rats at 0 days until 21 days after their offspring were born. We also preliminarily discussed the mechanism of BPA affecting hippocampal development. The aim of this study was to investigate the effect of BPA exposure during pregnancy on the hippocampal development and function in offspring.

## Materials and Methods

### Animal model

Thirty 10-week-old female adult Wistar rats weighing 200-220 g were fed adaptively for one week before being formally reared. According to body weight, the rats were randomly divided into three groups: 0 mg/kg/day group, 5 mg/kg/day group and 50 mg/kg/day group, with 10 female rats in each group. In each group, female rats were mated with normal male rats at 1:2, and gestational day 0 (GD0) was recorded as the first day of pregnancy. Starting from GD0, different concentrations of BPA ((CAS 80-05-7, (CH3)2C(C6H4OH)2, ≥99% purity) (Sigma Aldrich) was dissolved in ethanol (1% of final solution) and diluted with well-flushed tap water to defined concentrations) were gavage. 0 mg/kg/day group received water containing 1% ethanol (vehicle). Gastric administration lasted until the 21st day after the birth of the offspring (postnatal day 21, PND21). At PND 4, in order to obtain better nutrition from maternal rats and maintain normal growth and development, 8-10 offspring were retained in each litter to ensure the same sex ratio as possible. The main routes of BPA exposure were via the placenta in utero and via lactation. No rats died before the end of the experiment. This study was carried out in accordance with the principles of the Basel Declaration and recommendations of 2016PS312K, Ethics Committee of Shengjing Hospital of China Medical University. The protocol was approved by the Shengjing Hospital of China Medical University.

### Weight statistics

The weight of pregnant rats was recorded at the beginning, middle and late stages of pregnancy. The weight of newborn rats was recorded. At PND7, PND14 and PND21 days, cerebellum, left hippocampus, right hippocampus, spleen, thyroid and pancreas of offspring were weighed and recorded.

### Measurement of long-term synaptic plasticity in hippocampus

The rats were anesthetized with 20% uratan (6.5 ml/kg, ip) for 70-80 hrs and fixed on the stereotaxic apparatus of rats. Referring to the brain atlas of Paxions rats, bipolar stimulus electrodes were inserted into the pyramidal cell layer of CA3 area in the right hippocampus (coordinates: 4.0 mm after the anterior fontanel, 3.8 mm by the side, 4.0 mm deep subcortical). The recording electrodes were inserted into the pyramidal cell layer of CA1 area in the right hippocampus (coordinates: 4.0 mm after the anterior fontanel, 2.0 mm by the side, 2.0 mm deep subcortical). Population peak potentials were evoked by a single stimulus (intensity 7.5 V, wave width 0.1 ms). After 30 minutes of baseline recording, high frequency stimulation (100 Hz) with the same parameters was given for 5 seconds. After stimulation, the changes of synaptic plasticity in hippocampal CA3-CA1 region were observed. Monopulse test was used to examine the changes of the amplitude of population spike (PS) or the slope of field-excitatory postsynaptic potential (f-EPSP) induced by stimulation and to observe the duration of this change in order to analyze the effect of BPA on long-term synaptic plasticity in CA3-CA1 region of hippocampus in offspring. During the experiment, the stimulus parameters were kept consistent between groups and individuals.

### Immunofluorescence

At PND7, PND14 and PND21, anaesthetized offspring were exposed to heart by thoracotomy. Heart was perfused with 0.02% heparin saline of 50-100 ml. After blood clarification, 4% polyformaldehyde of 200-400 ml potassium phosphate buffer containing 0.1 M was perfused. When the limbs of rats were stiff, the brain was quickly removed from the skull and spent the night in 4% paraformaldehyde fixative. The fixed cerebrum was embedded in paraffin and coronal sections were made. The thickness of the cerebrum was 5 microns. After dewaxing to water, antigen was repaired, serum was dripped and blocked, and the first antibody was incubated overnight at 4 C. Re-warming at 37 for 30 minutes, PBS was soaked and then incubated with fluorescent antibody for 2 hours in the absence of light. PBS washed off the second antibody and dripped with anti-quenching sealing tablet containing DAPI. Typical photographs were observed and collected under Nikon 90I immunofluorescence microscope.

### TUNEL assay

Tissue sections were placed in a dyeing vat and washed twice with xylene for 5 min each time. Wash it twice with absolute ethanol for 3 min each time. Wash with 95% and 75% ethanol for 3 min each time. Wash with PBS for 5 min, add protease K solution (20ug/ml), and hydrolyze at room temperature for 15 min. Wash 4 times with distilled water, 2 min each time. The PBS reaction with 2% hydrogen peroxide lasted for 5 min. Wash twice with PBS for 5 min each time. Two drops of TdT enzyme buffer were added to the slices and placed at room temperature for 5 min. TdT enzyme solution was dripped onto the slices and reacted at 37 °C for 1 h in a wet box. The slices were placed in the dyeing vat, and the washing and termination buffer which had been preheated to 37 °C was added. The slices were kept at 37 °C for 30 min. After washing 3 times with PBS, 5 min each time, two drops of anti-digoxin antibody labeled with peroxidase were directly added to the slices and reacted at room temperature for 30 min in a wet box. Wash 4 times with PBS, 5 min each time. Fresh 0.05% DAB solution was added and coloured at room temperature for 3 min. Wash 4 times with distilled water, 1 minute each time for the first 3 times. At room temperature, methyl green was used for re-dyeing for 10 min. Wash it three times with distilled water. Xylene was dehydrated three times, 2 min each time. After sealing and drying, the experimental results were observed and recorded under optical microscope.

### Reverse transcription and quantitative real-time PCR

Total RNA of tissues after treating with different factors for 24 h were isolated by TRIzol (Invitrogen, USA) according to the manufacturer's protocol. Complementary DNA was synthesized by reverse transcription of total RNA using a RT reaction kit (Promega, USA). Quantitative real-time PCR was used to determine levels of mRNAs using the Assays-on-Demand Taqman Gene Expression Assays (Applied Biosystems, Foster City, CA, USA) according to the procedure previously described. Results were conducted in triplicate in at least five independent experiments. SYBR Premix Ex Taq (TaKaRa, Japan) was used as a DNA-specific fluorescent dye. Primer sequences were synthesized as shown in table 1.

All the reactions were repeated at least three times. Gene expression levels were calculated relative to GAPDH (glyceraldehyde-3-phosphate dehydrogenase) by using Stratagene Mx 3000P software.

### Western blot

Ultrasound cell crusher was used to homogenize and incubate on ice for 1 hour. The supernatant was centrifuged at 13,000 g 4 C for 10 min and then removed into the new tube. The protein concentration of each sample was determined by BCA kit. A protein mixture containing 30 UG was added to each swimming lane. 10% SDS-PAGE was separated by electrophoresis (100 V, 1.5 h) and transferred to PVDF membrane (100 V, 1 h). 5% skimmed milk powder was sealed at room temperature for 1 hour. After washing the film, the first resistance was added to incubate overnight. The next day, the second antibody was incubated at room temperature for 1 hour, and ECL chemiluminescence reaction was carried out. GAPDH was used as internal reference.

### Statistical analysis

Data were analyzed by SPSS17.0 software. Single factor analysis of variance (ANOVA) was used to compare the results of Western Blot. When the difference was statistically significant, SNK method was used to compare the results between groups. The electrophysiological data were analyzed by Kruskal-Wallis H test. *P* < 0.05 was statistically significant. All experiments were repeated three times and all data from three independent experiments were expressed as mean ± SD.

## Results

### Effects of BPA on physiological function of pregnant and offspring rats

**Figure 1:**
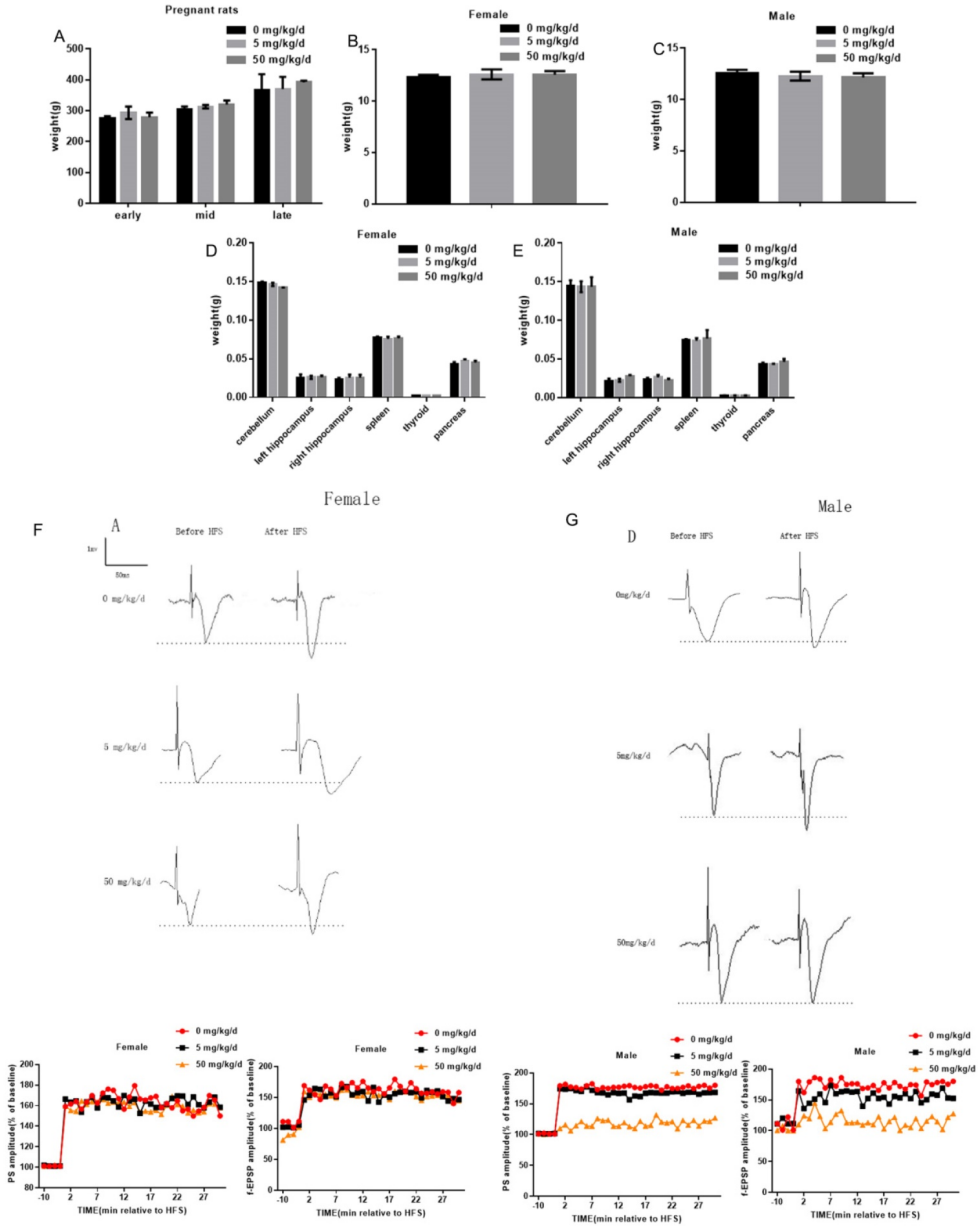
Effects of BPA on physiological function of pregnant and offspring rats. (**A**) The weight of pregnant rats was measured at different periods. Data are shown as mean ± SEM. N=8. (**B, C**) The weight of female offspring and male offspring were measured at PND7, PND14 and PND21 respectively after birth. Data are shown as mean ± SEM. N=8. (**D, E**) At PND7, PND14 and PND21 days, cerebellum, left hippocampus, right hippocampus, spleen, thyroid and pancreas of offspring were weighed. Data are shown as mean ± SEM. N=8. (**F, G**) Measurement of long-term synaptic plasticity in hippocampus. LTP induction was recorded for at least 30 min. Based on the pooled data, the means of the population spike (PS) amplitude and field-excitatory postsynaptic potential (f-EPSP) slope were expressed as a percentage of the corresponding pre-stimulation control. N=8.

In this study, 5 mg/kg/d BPA and 50 mg/kg/d BPA were applied to pregnant rats respectively, and the weight of pregnant rats was measured at different periods. The results showed that BPA did not affect the body weight of pregnant rats (Figure 1A). To determine whether BPA exposure can affect the biological behavior of offspring by inhibiting organ development, we weighed and recorded the weight of cerebellum, left hippocampus, right hippocampus, spleen, thyroid and pancreas of offspring. The results showed that BPA had no significant effect on the weight of offspring (Figure 1B, C). The results showed that BPA had no significant effect on organ weight of offspring (Figure 1D, E). Typical field potential changes in pyramidal cell layer of hippocampal CA1 region were observed before and after exposure to different concentrations of BPA. The results showed that LTP did not occur in female offspring and male offspring exposed to BPA at low concentrations. After high concentration BPA exposure, the increase of PS amplitude and f-EPSP slope in hippocampal CA1 area of male offspring was lower than that of control group, and the difference was statistically significant. It is suggested that high concentration of BPA exposure may cause mild LTP damage in male offspring (Figure 1F, G).

### Effects of BPA on the proliferation of hippocampal neurons in offspring rats

**Figure 2:**
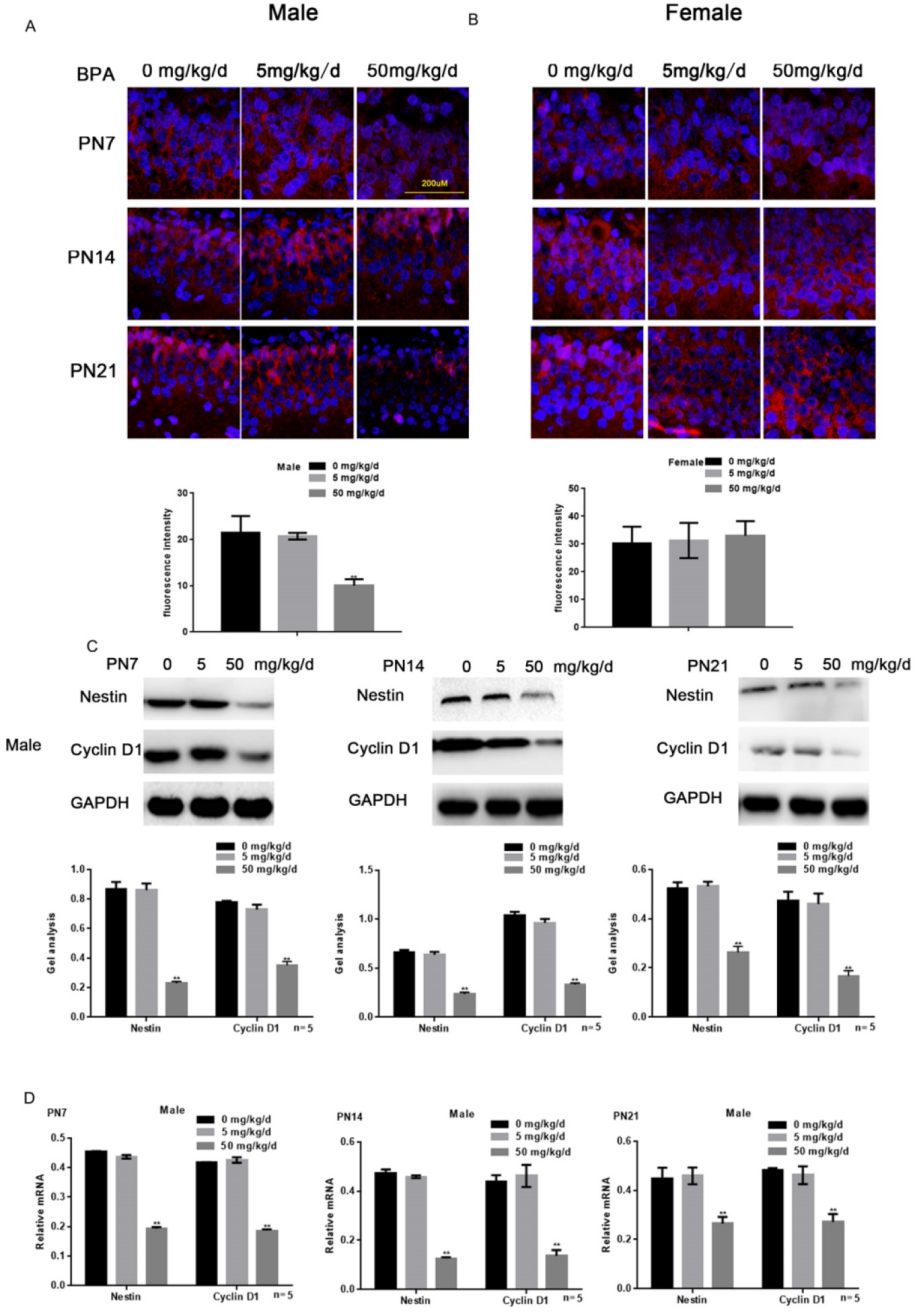
** Effects of BPA on the proliferation of hippocampal neurons in offspring rats.** (**A**) Immunofluorescence staining results showed the expression of Nestin in hippocampal DG region neurons of male offspring rats. N=5. (**B**) Immunofluorescence staining results showed the expression of Nestin in hippocampal DG region neurons of female offspring rats. N=5. (**C, D**) Western blot and real-time PCR showed that the expression of Nestin and Cyclin D1 in the hippocampus of male offspring. Data are shown as mean ± SEM. ** P < 0.05 vs. control group. N=5. (**E, F**) Western blot and real-time PCR showed that the expression of Nestin and Cyclin D1 in the hippocampus of female offspring. Data are shown as mean ± SEM. N=5.

Immunofluorescence staining results showed that high concentration of BPA could inhibit the expression of Nestin in neurons of hippocampal dentate gyrus (DG) region in male offspring rats (Figure 2A). But there was no significant change in female offspring (Figure 2B). The results of Western blot and real-time PCR showed that the expression of Nestin and Cyclin D1 in the hippocampus of male offspring was significantly down-regulated by BPA at 50 mg/kg/d, however not at 5 mg/kg/d BPA (Figure 2C, D). The results of real-time PCR also showed that Nestin expression in PND14 and 21 was slightly down regulated compared with that in PND7. Furthermore, BPA had no significant effect on the expression of Nestin and Cyclin D1 in the hippocampus of female offspring (Figure 2E, F).

### Effects of BPA on the apoptosis of hippocampal neurons in offspring rats

**Figure 3:**
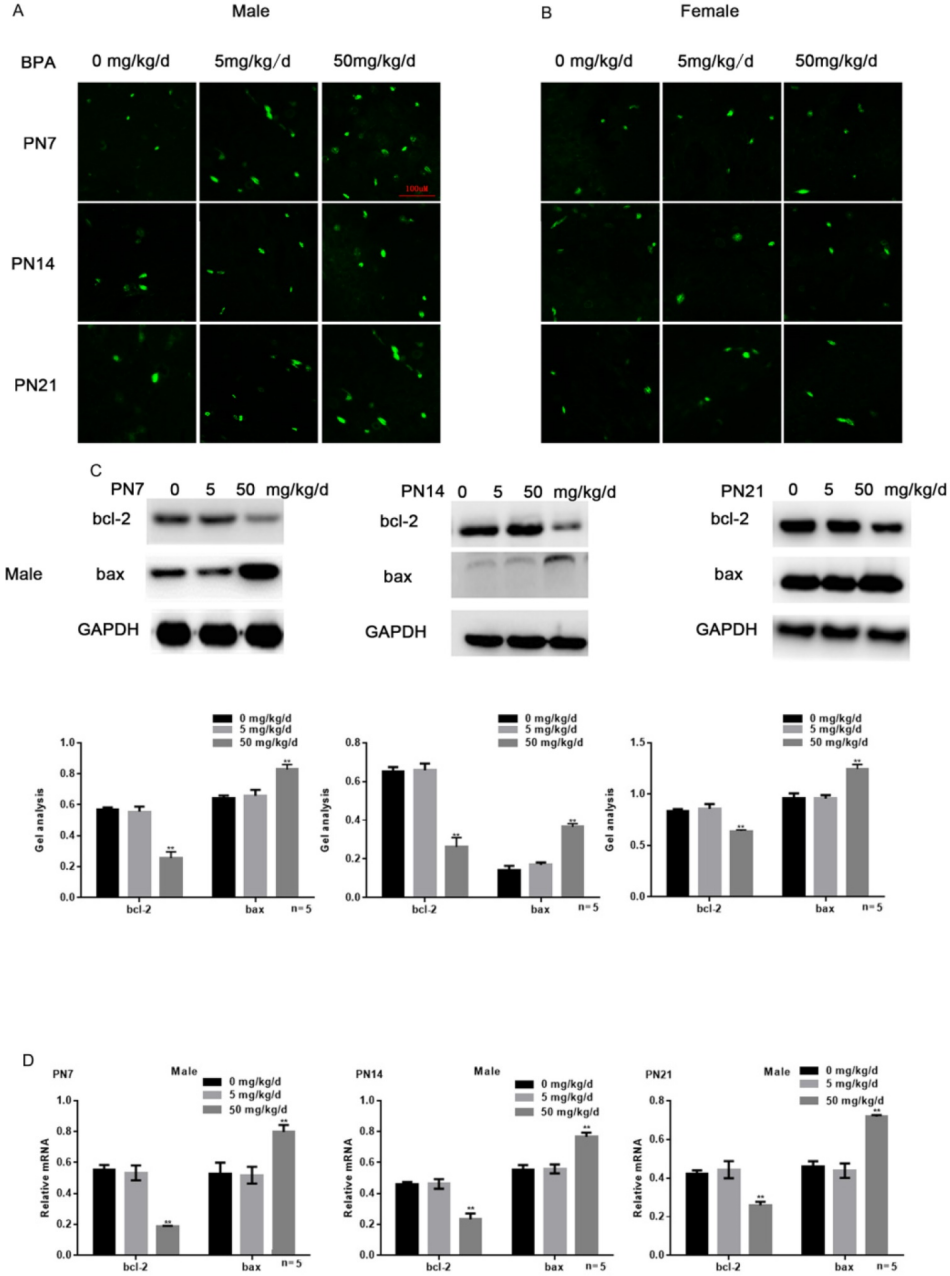

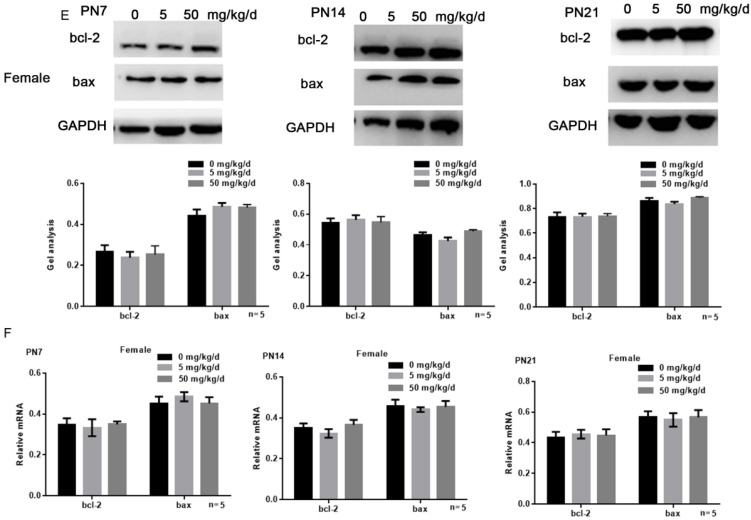
** Effects of BPA on the apoptosis of hippocampal neurons in offspring rats.** (**A**) Tunel assay results showed the apoptosis of hippocampal CA1 region neurons of male offspring rats. N=5. (**B**) Tunel assay results showed the apoptosis of hippocampal CA1 region neurons of female offspring rats. N=5. (**C, D**) Western blot and real-time PCR showed that the expression of bcl-2 and bax in the hippocampus of male offspring. Data are shown as mean ± SEM. ** P < 0.05 vs. control group. N=5. (**E, F**) Western blot and real-time PCR showed that the expression of bcl-2 and bax in the hippocampus of female offspring. Data are shown as mean ± SEM. N=5.

Tunel assay results showed that high concentration of BPA could promote the apoptosis of CA1 hippocampal neurons of male offspring rats (Figure 3A), but had little effect on female offspring (Figure 3B). The results of Western blot and real-time PCR showed that the expression of bcl-2 was down regulated and the expression of bax was up regulated in the hippocampus of male offspring by BPA at 50 mg/kg/d, however not at 5 mg/kg/d BPA (Figure 3C, D). For female offspring, BPA had no significant effect on the expression of bcl-2 and bax in the hippocampus (Figure 3E, F).

### Effects of BPA on the expression of RhoA/Rac1 in hippocampal neurons in offspring rats

**Figure 4:**
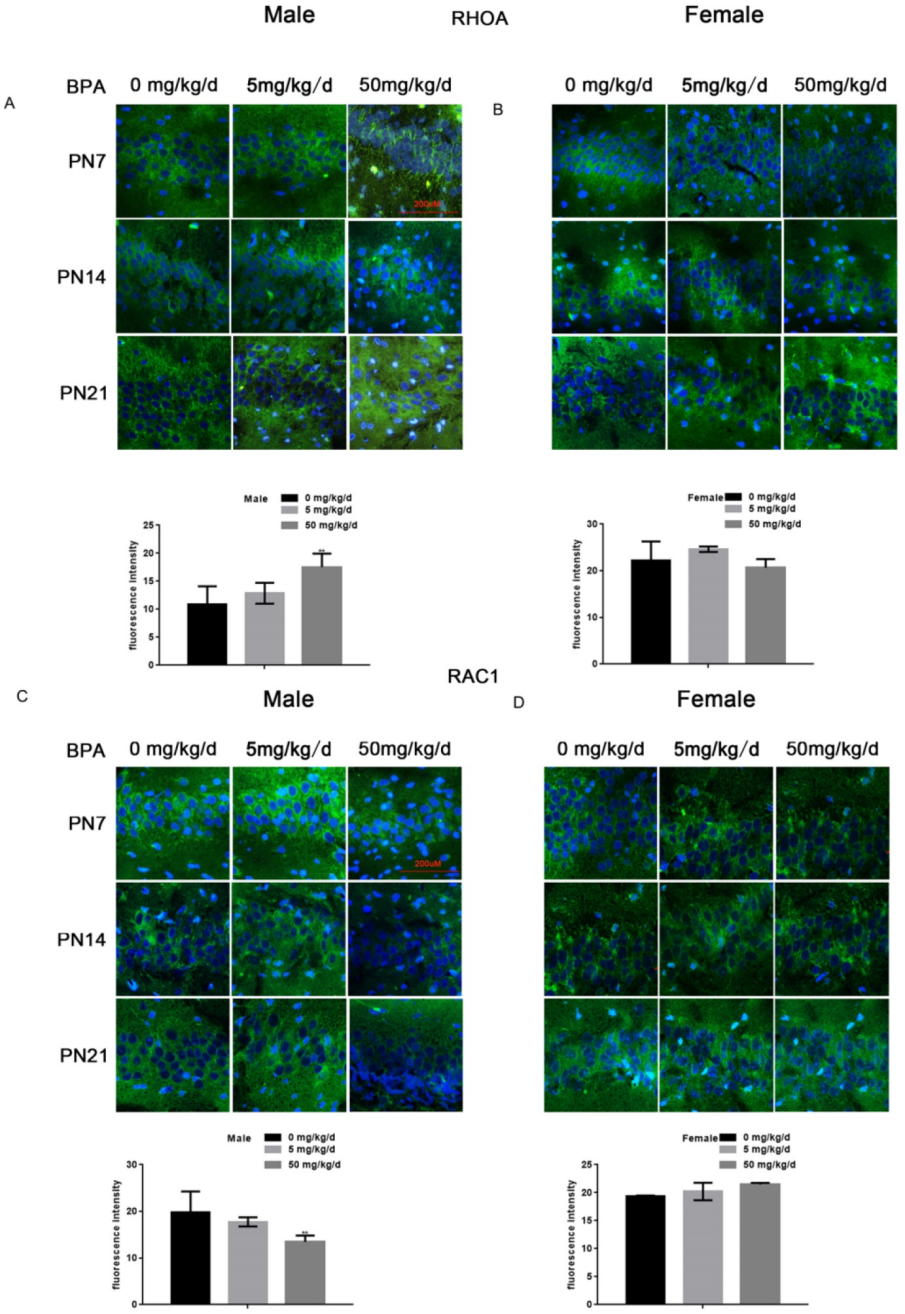
** Effects of BPA on the expression of RhoA/Rac1 in hippocampal neurons in offspring rats.** (**A, B**) Immunofluorescence staining results showed the expression of RhoA in CA1 hippocampal neurons of offspring rats. N=5. (**C, D**) Immunofluorescence staining results showed the expression of Rac1 in hippocampal CA1 region neurons of offspring rats. N=5.

**Figure 5:**
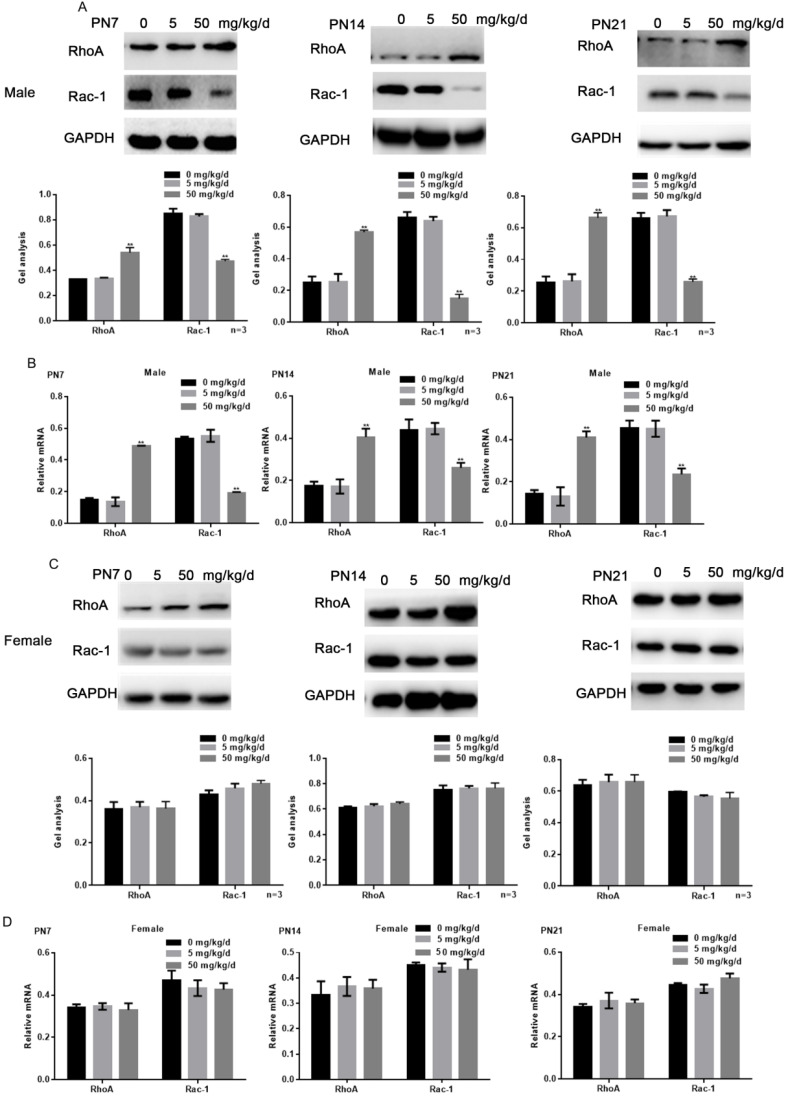
** Effects of BPA on the expression of RhoA/Rac1 in hippocampal neurons in offspring rats.** (**A, B**) Western blot and real-time PCR showed that the expression of RhoA and Rac1 in the hippocampus of male offspring. Data are shown as mean ± SEM. ** P < 0.05 vs. control group. N=5 (**C, D**) Western blot and real-time PCR showed that the expression of RhoA and Rac1 in the hippocampus of female offspring. Data are shown as mean ± SEM. N=5.

Because RhoA and Rac1 play an important role in hippocampal nerve development, we examined the effects of BPA on RhoA and Rac1 in hippocampal neurons. The results of fluorescence staining showed that BPA at 50 mg/kg/d could significantly up-regulate the expression of RhoA and down-regulate the expression of Rac1 in the hippocampal CA1 region of male offspring (Figure 4A, C). However, the effect of low concentration BPA was not significant, and the effect of BPA on female offspring was not significant (Figure 4A-D). The results of Western blot and real-time PCR showed that the expression of RhoA was up regulated and the expression of Rac1 was down regulated in the hippocampus of male offspring by BPA at 50 mg/kg/d, however not at 5 mg/kg/dBPA (Figure 5A, B). The results of Western blot and real-time PCR showed that BPA had no significant effect on the expression of RhoA and Rac1 in the hippocampus of female offspring (Figure 5C, D).

## Discussion

The Centers for Disease Control and prevention reported measurable levels of BPA in urine samples from more than 90 percent of the U.S. population 15. BPA shows the endocrine interference effect by interacting with estrogen receptor α/β, estrogen related receptor γ, androgen receptor and thyroid hormone receptor 4, 16, 17. In addition, epidemiological studies have shown that BPA exposure may be related to changes in hormone levels, impairment of ovarian and uterine functions, and decreased sperm quality 18, 19. The latest data of experimental studies showed that bisphenol A exposure had adverse effects on oocyte quality, sperm production and quality, testicular cells, hormone level, ovarian function and uterine morphology in animal models 20. Studies have also shown that maternal BPA exposure during childbirth can affect offspring learning and memory ability 21. However, the potential mechanism of Bisphenol A related to brain development still needs to be further explored.

In our study, we treated pregnant rats with different concentrations of BPA at the time of conception. Compared with the control group, BPA did not affect the weight of pregnant rats. Then, we continued to give BPA to the pregnant rats until postnatal 21 days. It was found that different concentrations of BPA did not affect the body weight of offspring, nor did they significantly regulate the weight of cerebellum, left hippocampus, right hippocampus, spleen, thyroid and pancreas of offspring. It has been reported that toxic exposure patterns can lead to cytotoxicity and cell apoptosis, affecting astrocytes and neurons in the hippocampus, but not necessarily affecting the weight of rats^22^, which is similar to our research. *In vivo* neuroelectrophysiological experiments showed that the increase of f-EPSP slope and PS amplitude in hippocampal CA1 region of male rats in BPA group was lower than that in control group after high frequency stimulation. It is suggested that BPA can cause slight damage to long-term potentiation in hippocampal CA1 region of rat offspring. LTP is an important synaptic model for studying the process of learning and memory. So we speculated that BPA may affect the hippocampal function.

Subsequent electrophysiological results showed that with the increase of BPA exposure dose, the average enhancement rate of male rats after high-frequency stimulation decreased. Although there was no significant difference between low-dose group and control group, the high-dose group was significantly lower than the control group. But the study also showed that BPA had no significant effect on female rats. The results showed that BPA exposure could decrease the spinous synaptic density of hippocampus in ovariectomized female rats, but BPA combined with estrogen could not. Studies showed that BPA concentrations were positively correlated with both estrogenic and anti-androgenic activities and BPA embryonic exposure increased the expression of estrogen receptor 23, 24. In our opinion all of these were inferred that the effect of BPA was related to the content of estrogen. Estrogen can offset the inhibition of neurodevelopment caused by BPA to some extent. In this study, we concluded that high concentration of BPA can inhibit the neurodevelopment of male offspring, which is consistent with previous studies.

Most mammals, including humans, develop mature neurons in the dentate gyrus of the hippocampus through the proliferation, migration and differentiation of stem cells 25. The expression of important biomarkers in the main structure of the nervous system responsible for neurogenesis in the individual, and in the choroid plexus, was demonstrated by nestin 26. It is pointed out that the neurotoxic effect of drugs on nerve cells can be reflected by the regulation of proliferation and apoptosis of nerve cells 27. It was pointed out that promoting the proliferation of mouse hippocampal DG cells, increasing the density of immature neurons and the expression of nestin protein could improve the learning and memory ability of rats. So we observed nestin and Cyclin D1 expressions in DG region 28. Our results showed that high concentration of BPA could inhibit the expression of Nestin and the levels of Nestin and Cyclin D1 in the hippocampus of male offspring. Moreover, it was indicated that high concentration of BPA could promote apoptosis in the hippocampus of male offspring. So, we speculated that the decreased proliferation and increased apoptosis of neurons induced by maternal BPA exposure might contribute to impairments of hippocampal function.

RhoA signaling pathway was involved in the inhibition of dendritic development in both cultured neurons and developing cortical neurons 11. The up-regulation of RhoA in hippocampus of male offspring after high concentration BPA treatment was observed 29. On the contrary, Rac1 plays an important role in synaptic growth and maturation 30. It has been confirmed that Rac1 plays an important role in neuronal morphological development and neurotransmitter release 31. The level of Rac1/RhoA increased in repairing nerve injury in rats and the dendritic length and complexity and the density of dendritic spines markedly increased in the hippocampal CA1 and the dentate gyrus 32. Our study showed that maternal BPA exposure can increase the expression of RhoA and reduce the expression of Rac1 in hippocampus of male pups. It is speculated that the alterations of Rac1/RhoA following BPA exposure might lead dendritic damage, which might result in the impairments of hippocampal function. In addition, RhoA and Rac1 can also regulate the biological function of cells by activating some other effector proteins. Nestin, Cyclin D1, Bcl-2 and Bax can all act as effector proteins downstream of RhoA and Rac1 25. So, we speculated that the effects of BPA on the hippocampus of male offspring might be partly mediated by the regulation of RhoA and Rac1. Researchers reported that BPA-exposed male offspring not only spent more time in exploring the familiar object at the highest dose than the control, but also displayed a significantly decreased the object recognition index at the doses of 0, 5 and 50 mg/kg BW/day. In this study, no behavioral tests were conducted before the sacrifice of offspring^33^. Others found out that treatments significantly abrogated spatial learning and ability in males offspring^34^. We lack behavioral data on the memory of young rats. We expect to supplement it in subsequent experiments.

In summary, our study indicated that maternal exposure to high concentration of BPA could impair the hippocampal function of male offspring by affecting the growth and apoptosis of hippocampal neurons, which might be attributed to the abnormal regulation of RhoA and Rac1.

## Figures and Tables

**Table 1:** Primer sequences

Name	Forward primer (5'->3')	Reverse primer (5'->3')
RhoA	AAACTGGTGATTGTTGGTGATGG	TTTCACCGGCTCCTGCTTTC
Rac1	GCGGCCATTTCCTGTTTCTC	CGGATAGGATAGGGGGCGTA
Nestin	GTAGCTCCCAGAGAGGGGAA	CTCTAGAGGGCCAGGGACTT
Cyclin D1	CCGAGGAGCTGCTGCAAATGGAG	TGAAATCGTGCGGGGTCATTGCG
bcl-2	GGTGAACTGGGGGAGGATTG	GGCAGGCATGTTGACTTCAC
bax	AGCTGAGCGAGTGTCTCAAG	GTCCAATGTCCAGCCCATGA
GAPDH	AGAAGGCTGGGGCTCATTTG	AGGGGCCATCCACAGTCTTC
